# Antiparasitic and anti-inflammatory activities of ß-lapachone-derived naphthoimidazoles in experimental acute *Trypanosoma cruzi* infection

**DOI:** 10.1590/0074-02760190389

**Published:** 2020-02-14

**Authors:** Cynthia M Cascabulho, Marcelo Meuser-Batista, Kelly Cristina G de Moura, Maria do Carmo Pinto, Thabata Lopes Alberto Duque, Kelly C Demarque, Ana Carolina Ramos Guimarães, Pedro Paulo de Abreu Manso, Marcelo Pelajo-Machado, Gabriel M Oliveira, Solange L De Castro, Rubem FS Menna-Barreto

**Affiliations:** 1 Fundação Oswaldo Cruz-Fiocruz, Instituto Oswaldo Cruz, Laboratório de Inovações em Terapias, Ensino e Bioprodutos, Rio de Janeiro, RJ, Brasil; 2 Fundação Oswaldo Cruz-Fiocruz, Instituto Fernandes Figueira, Departamento de Anatomia Patológica e Citopatologia, Laboratório de Patologia Molecular, Rio de Janeiro, RJ, Brasil; 3 Universidade Federal do Rio de Janeiro, Instituto de Pesquisa em Produtos Naturais, Rio de Janeiro, RJ, Brasil; 4 Fundação Oswaldo Cruz-Fiocruz, Instituto Oswaldo Cruz, Laboratório de Biologia Celular, Rio de Janeiro, RJ, Brasil; 5 Fundação Oswaldo Cruz-Fiocruz, Instituto Oswaldo Cruz, Laboratório de Genômica Funcional e Bioinformática, Rio de Janeiro, RJ, Brasil; 6 Fundação Oswaldo Cruz-Fiocruz, Instituto Oswaldo Cruz, Laboratório de Patologia, Rio de Janeiro, RJ, Brasil

**Keywords:** Trypanosoma cruzi, Chagas disease, chemotherapy, naphthoimidazoles, heart, cardiomyopathy

## Abstract

**BACKGROUND:**

Chagas disease, which is caused by the protozoan *Trypanosoma cruzi*, is endemic to Latin America and mainly affects low-income populations. Chemotherapy is based on two nitrocompounds, but their reduced efficacy encourages the continuous search for alternative drugs. Our group has characterised the trypanocidal effect of naphthoquinones and their derivatives, with naphthoimidazoles derived from β-lapachone (N1, N2 and N3) being the most active *in vitro*.

**OBJECTIVES:**

In the present work, the effects of N1, N2 and N3 on acutely infected mice were investigated.

**METHODS:**

*in vivo* activity of the compounds was assessed by parasitological, biochemical, histopathological, immunophenotypical, electrocardiographic (ECG) and behavioral analyses.

**FINDINGS:**

Naphthoimidazoles led to a decrease in parasitaemia (8 dpi) by reducing the number of bloodstream trypomastigotes by 25-50% but not by reducing mortality. N1 protected mice from heart injury (15 dpi) by decreasing inflammation. Bradycardia was also partially reversed after treatment with N1 and N2. Furthermore, the three compounds did not reverse hepatic and renal lesions or promote the improvement of other evaluated parameters.

**MAIN CONCLUSION:**

N1 showed moderate trypanocidal and promising immunomodulatory activities, and its use in combination with benznidazole and/or anti-arrhythmic drugs as well as the efficacy of its alternative formulations must be investigated in the near future.

Chagas disease, which is caused by the protozoan *Trypanosoma cruzi*, is endemic to Latin America and affects approximately 6 million individuals.^1^ This disease, which is classically associated with rural populations, underwent an urbanisation process in Latin American cities and later in the USA, European countries, Japan and Australia.^2^ Although vectorial (haematophagous triatomine) and transfusional transmission have steadily declined due to the success of control programs, congenital and oral transmission have become important sources of new cases.^3^ Chagas disease is characterised by acute and chronic phases. The acute phase is defined by patent parasitaemia and is frequently asymptomatic. The chronic phase can be divided into three clinical forms: the indeterminate or asymptomatic, cardiac (30% of patients) and digestive forms (megasyndromes). Only two drugs, 2-nitroimidazole benznidazole (Bz) and 5-nitrofuran nifurtimox, are licensed for the treatment of Chagas disease. Both drugs have shown successful results in the acute phase, but their effectiveness decreases with advancement of the infection.^4^ Severe adverse reactions and limited efficacy in the late chronic phase justify the urgent need for new drugs/combinations to treat chagasic patients.^5^

The impact of natural products on drug discovery is considerable, not only for cancer but also for parasitic infections. Naphthoquinones are currently used in medicinal chemistry to synthesize derivatives with potential activity against cancer, fungi, bacteria and pathogenic protozoa.^6^ The bioactivity of naphthoquinones involves the generation of oxidative stress via the production of reactive oxygen species (ROS) and the alkylation of nucleophilic biomolecules.^7^

The activity of naphthoquinones and their derivatives against *T. cruzi* has been intensively studied by different research groups.^8^ For the past 20 years, while working on experimental chemotherapy for Chagas disease, our group, originally under the leadership of Dr Antonio Ventura Pinto, has been investigating derivatives of lapachones to explore their reactivity towards nucleophilic reagents.^8^ ^,^ ^9^ The reaction of β-lapachone with aldehydes leads to the insertion of an imidazole nucleus into naphthoimidazoles. Among a series of 34 derivatives, N1, N2 and N3 displayed the highest activity against *T. cruzi* trypomastigotes, with their activity being 3- to 18-fold higher than that of the standard drug Bz.^9^ ^,^ ^10^ ^,^ ^11^ Subsequent studies pointed to the parasite mitochondrion and the autophagic pathway as the primary targets of these compounds.^12^ ^,^ ^13^ Through proteomic approaches, we identified a great number of mitochondrial proteins that are differentially expressed in naphthoimidazole-treated trypomastigotes and epimastigotes.^14^ ^,^ ^15^ Recently, the association between the trypanocidal activity of these naphthoimidazoles and mitochondrial oxidative stress was demonstrated *in vitro*. Treated parasites showed high trypanothione reductase activity and were positioned in pockets of this enzyme, which was in line with the increase in trypanothione synthetase activity that was found in a previous proteomic study.^16^

## MATERIALS AND METHODS

**Fig. 1:**
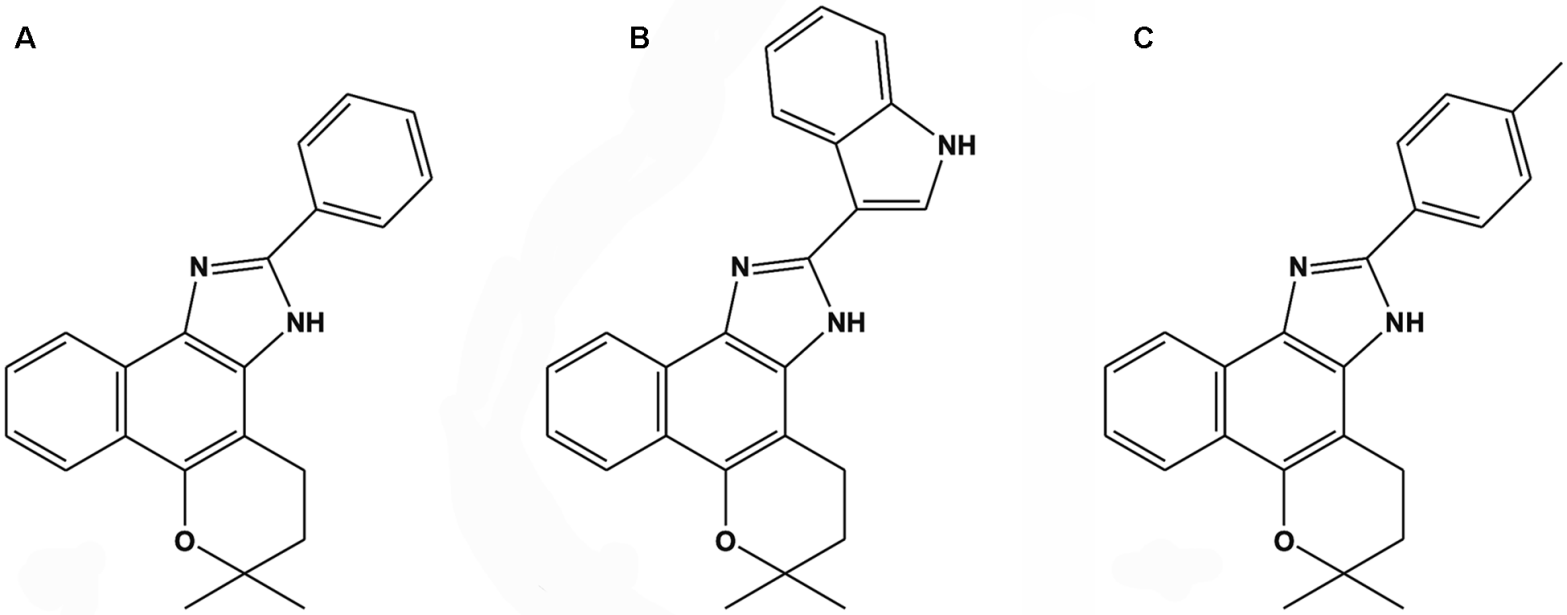
chemical structures of the β-lapachone-derived naphthoimidazoles: (A) N1, (B) N2 and (C) N3.

*Naphthoimidazole synthesis* - The reaction of β-lapachone with benzaldehyde, indolylaldehyde or 4-methylbenzaldehyde in the presence of ammonium acetate resulted in N1 (4,5-dihydro-6,6-dimethyl-6H-2-(phenyl)-pyran[b-4,3]naphth[1,2-d]imidazole), N2 (4,5-dihydro-6,6-dimethyl-6H-2-(3´-indolyl)-pyran[b-4,3]naphth[1,2-d] imidazole) or N3 (4,5-dihydro-6,6-dimethyl-6H-2-(4´-methylphenyl)-pyran[b-4,3]naphth[1,2-d]imidazole), respectively, as previously reported (Fig. 1).^9^ ^,^ ^10^ ^,^ ^11^ The stock solutions of the three naphthoimidazoles were prepared in water containing 20% Tween-80, which was vortexed and sonicated immediately before administration. Preliminary data revealed that the presence of vehicle did not alter the course of infection.

*Mice* - The use of male outbred stock Swiss Webster mice (weight: 18-20 g) and the experimental procedures were performed in accordance with Brazilian Law 11.794/2008 and the regulations of the National Council of Animal Experimentation Control (CONCEA). The mice were housed with a maximum of six individuals per cagein a specific-pathogen-free (SPF) room at 20 to 22ºC under a 12/12 h light/dark cycle with 50 to 60% humidity and provided sterilised water and chow *ad libitum*. On the 15th day post infection (dpi) with *T. cruzi*, a group of animals was euthanised using an overdose of CO_2_ followed by cervical dislocation. To preserve animal welfare (due to parasite infection and/or compound administration), the daily observation was supervised by a PhD veterinary doctor with the aim of avoiding unnecessary animal suffering and pain. Animals were humanely euthanised using an overdose of CO_2_ followed by cervical dislocation whenever early endpoints due to animal suffering became necessary because of motor disturbance, a lack of exploratory activity and/or a moribund condition.

*Parasites, infection and treatment* - *T. cruzi* bloodstream trypomastigotes (Y strain) (10^4^ parasites/mice) were inoculated by an intraperitoneal route (i.p.). The experimental groups (N = 8 each) were as follows: noninfected and nontreated control, infected and nontreated control, infected + 100 mg/kg N1 (**N1**), infected + 100 mg/kg N2 (**N2**) and infected + 100 mg/kg N3 (**N3**). The treatment was performed by gavage every other day beginning on the seventh day post infection (dpi 7). The noninfected and infected groups received the same volume of the vehicle.

*Parasitaemia, body weight and mortality* - Parasitaemia was individually checked by direct microscopy by using the Pizzi-Brener method.^17^ Briefly, 5 μL of blood was added between a slide and a coverslip, and 50 fields were counted randomly; the concentration of the parasites was calculated based on a specific factor for a given microscope. The body weight was evaluated weekly between0 and 34 dpi. The cumulative mortality was noted daily, and the percent survival was calculated at dpi 37.

*Evaluation of the behavioral/clinical parameters* - During the infection course, the physical/clinical aspects and food consumption were monitored daily. The following parameters were analysed: body posture; skin integrity (injury and/or peeling); fur appearance (piloerection, dull fur, focal or diffuse alopecia); infestation by ectoparasites and the presence of clinical signs associated with secondary infections, such as dermatitis and conjunctivitis. Food and water consumption were measured daily (beginning one week prior to the infection) by calculating the differences between the weight/volume offered (250 g/250 mL) and the amounts left in each cage after 24 h. The individual consumption amounts were estimated using previously reported formulae.^18^

Cons_total_ = weight/volume added - weight/volume after 24 h.

Cons_ind_ = Cons_total_/number of animals per cage.

*Motor and exploratory activity studies* - To analyse the spontaneous activity of individual mice, each animal was monitored by the video-tracking tool NoldusEthoVisionXT6 (Noldus Information Technology, Leesburg, Netherlands). The arena was defined as twelve rectangles that were divided into lateral and central areas and calibrated to contain equal areas to ensure the consistency of the parameters. The (a) motor activity, which was defined as the covered distance (cm), (b) average velocity (cm/s), and (c) exploratory activity, which was defined as the frequency of travel to the central region (number of events) per 30 min period, were measured daily between 0 and 30 dpi.^19^

*Biochemical analysis* - Serum levels of aspartate aminotransferase (AST), alanine aminotransferase (ALT), urea and cardiac isoform of creatine kinase (CK-MB) were measured as indicators of hepatic (AST and ALT), renal (urea) and cardiac (CK-MB) function. Blood samples were collected from tail snips at 0 and 15 dpi using commercially available kits and analysed according to the manufacturer’s instructions (LabTest Laboratory, MG, Brazil).

*Absorption, distribution, metabolism, and excretion (ADME) analysis* - The chemical structures of the three compounds were redesigned using Marvin JS (https://docs.chemaxon.com/display/docs/Marvin+JS) and converted to MOL files. The Swiss ADME tool was used to calculate physicochemical descriptors as well as to predict Lipophilicity and water solubility.^20^

*Noninvasive blood pressure analysis* - Before we carried out the blood pressure data evaluation, the mice were manipulated daily and adapted for seven days, and a tail sphygmomanometer was fitted for three consecutive readings until stabilisation. Blood pressure was individually recorded at 0, 6, 9 and 15 dpi using an LE 5001 Pressure Meter® (PanLab Instruments, Barcelona, Spain) to evaluate caudal artery pressure in nonsedated animals. The values of the systolic (SP), diastolic (DP) and mean (MP) pressure were calculated as indicated by the manufacturer.^21^

*Electrocardiographic (ECG) analysis* - All animals were tranquilised with diazepan (5 mg/kg, i.p. route), and transducers were carefully placed under the skin in accordance with the chosen preferential derivation (DII). The traces were recorded using a digital system (Power Lab 2/20) connected to a bioamplifier at 2 mV for 1 s (PanLab Instruments). The filters were standardised between 0,1 and 100 Hz, and the traces were analysed using Scope Software for Windows V3.6.10 (PanLab Instruments). We measured the heart rate (bpm: beats per minute) and the duration of the PR, QRS, and QT intervals and the P wave (milliseconds) at 0, 7 and 14 dpi.^24^ The relationship between the QT interval and the RR interval was individually assessed. To obtain physiologically relevant values for the heart rate-corrected QT interval (QTc) in units of time rather than as time to a power not equal to 1, the observed RR interval (RR_0_) was first expressed as a unitless multiple of 100 ms to obtain the normalised RR interval (RR_100_ = RR_0_/100ms). Next, the value of the exponent (*y*) in the formula QT_0_ = QTc x RR^*y*^_100_ was determined, where QT_0_ is the observed QT and both QT and QTc are in milliseconds. By determining the natural logarithm of each side of the formula (QT_0_) = In (QTc) + *y*ln (RR_100_), the slope of the linear relationship between the log-transformed QT and RR_100_ thus defined the exponent to which the RR interval ratio should be raised to correct the QT for the heart rate.^22^

*Histopathological analysis* - At 15 dpi, the animals were euthanised by following Brazilian ethical guidelines, and the heart, liver and spleen were collected, sectioned, and fixed in 10% buffered formalin. All fragments were processed according to standard histological techniques for paraffin embedding. Sections (5 μM thick) were stained with hematoxylin and eosin to perform the comparative morphological analyses. For cardiac tissue, at least two duplicate sections were evaluated per animal, and approximately four sections per animal were evaluated for each organ. In the case of hepatic tissue, three areas of each organ were selected, which totaled approximately six sections per animal.

*Immunophenotypical analysis* - For the dissociation of cardiac muscle, the ventricles were collected and cut into fragments (1-2 mm thick) in ice-cold phosphate buffer saline (PBS). All fragments were transferred to a 0.1% solution of collagenase type IV (powder: 300 U/mg) (Sigma-Aldrich, St. Louis, USA) and subjected to seven or eight cycles of enzymatic digestion (15 min each) under gentle agitation at 37ºC. The isolated cells were centrifuged (400 g/10 min) and immediately transferred to ice-cold Roswell Park Memorial Institute (RPMI) 1640 medium supplemented with 10% foetal calf serum (Sigma-Aldrich), and the cells were maintained on ice until use. Alternatively, mouse splenocytes were obtained by mechanical dissociation, and the erythrocytes were lysed by hypotonic shock in RPMI 1640 culture medium (Gibco, Paisley, Great Britain) diluted 1:10 in water for 10 s. Cardiac cells and splenocytes were washed in cold RPMI and quantified using a Neubauer chamber.

For phenotypical labeling, the cells were incubated for 30 min at 4ºC in RPMI 1640 medium supplemented with 10% foetal calf serum and 10% inactivated normal sheep serum to block FcgR. All samples were incubated for 30 min at 4ºC with anti-CD3 PerCP, anti-CD4 FITC, anti-CD8 PECy-7,anti-CD49d PE, anti-CD62L APC-Cy7, anti-CD49e PE, and anti-LFA-1 PerCP, washed twice in RPMI 1640 medium and acquired with a Cyan ADP flow cytometer (Beckman Coulter, Houston, USA). Data analysis was performed using Summit software version 4.3 (Beckman Coulter).

*Statistical analysis* - Statistical analyses were performed using two-way ANOVA followed by a Bonferroni posttest or one-way ANOVA with Tukey´s multiple comparison test, and the results were considered significant when the p value was < 0.05.

*Ethics* - All animal experimental procedures were performed under a license (L-005/2017) approved by the Ethics Committee for Animal Use at the Oswaldo Cruz Institute (CEUA/IOC).

## RESULTS

**Fig. 2:**
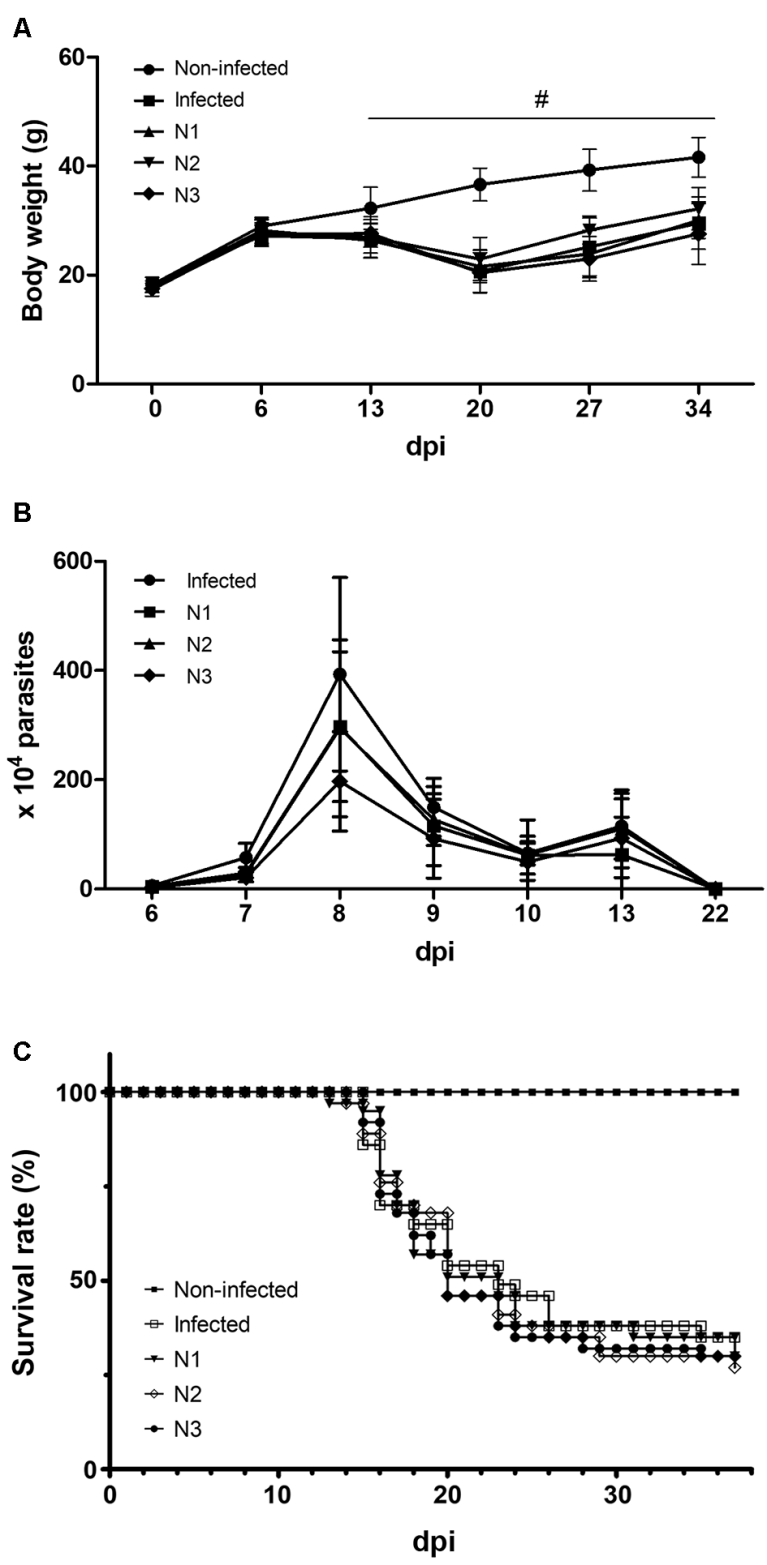
the effect of N1, N2 and N3 on the acute *Trypanosoma cruzi* infection course (Y strain). (A) Body weight. Significant differences between noninfected and infected animals were observed from 13 dpi to 34 dpi (p < 0.001). (B) Parasitaemia. Significant differences between the infected and treated groups were detected at 8 dpi (p < 0.05). (C) Mortality. Significant differences between the noninfected and infected (treated or untreated) were observed (p < 0.0001).

For the *in vivo* screening, naphthoimidazoles N1, N2 and N3 were administered orally to infected mice (five doses of 100 mg/kg every other day at 6-15 dpi), and the monitoring of parasitaemia, mortality and body weight were performed until 37 dpi (Fig. 2). The infection induced a significant loss of body weight at 13 dpi, and this phenotype was maintained until 34 dpi. At 20 dpi, the most dramatic decrease (43.4%) in body weight in all the infected animals was detected. However, no differences were observed between untreated and treated mice (Fig. 2A). In our acute model, the Y strain (inoculated with 10^4^ trypomastigotes/mice) showed the main peak of parasitaemia at 8 dpi, which was followed by a second less-representative peak at 13 dpi. At 8 dpi, the treatment with naphthoimidazoles led to a significant reduction in this parasitological parameter (p < 0.05). At this time point, N1 and N2 significantly decreased the number of bloodstream parasites by approximately 25%. However, the strongest effect on parasitaemia was observed after the administration of N3, which reduced by 50% the number of trypomastigotes in mouse blood at 8 dpi (Fig. 2B). The infection also led to a time-dependent increase in the mortality rates starting at 15 dpi, which increased up to 65% at 37 dpi. None of the three naphthoimidazoles protected the infected animals from death, and the mice showed similar mortality rates as the control mice (Fig. 2C). Additionally, the behavioral analysis showed no differences between the nontreated and treated groups in terms of velocity, motor and exploratory activities [Supplementary data (Fig. 1)] or even in terms of food or water consumption [Supplementary data (Fig. 1)]. The atypical behavior of N3-treated animals (i.e., intense spinning when being lifted by tail was frequently observed; data not shown) was suggestive of a vestibular disorder or neurological damage.

**Fig. 3:**
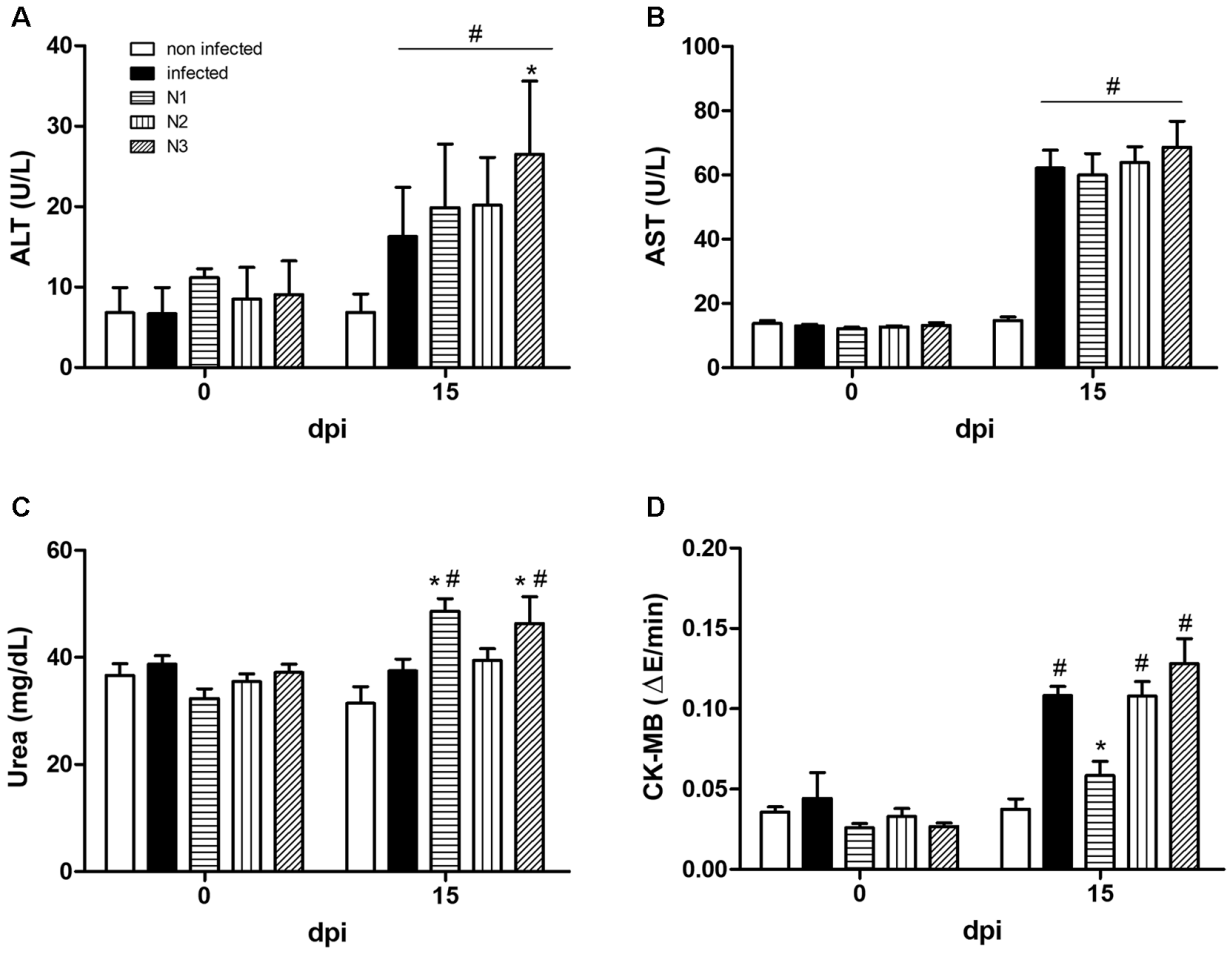
the effect of N1, N2 and N3 on the serum levels of biochemical markers in acute *Trypanosoma cruzi* infected mice. (A) alanine aminotransferase (ALT). (B) aspartate aminotransferase (AST). (C) Urea. (D) CK-MB. Serum samples for all groups were obtained at 0 and 15 dpi. Data were expressed as the means ± standard deviation (SD) from three independent experiments. #: comparisons between noninfected and infected (treated or untreated) groups (p < 0.05); *: comparisons between untreated and treated groups (p < 0.05).

Regarding the analysed biochemical markers, at 15 dpi, the infection caused hepatic (ALT and AST) and cardiac (CK-MB) damage, while no renal injury (urea) was detected. The levels of serum ALT, AST and CK-MB in infected animals were 2.4-, 4.2- and 2.8-fold higher than those in noninfected animals (Fig. 3). Naphthoimidazoles did not reverse the hepatic lesions caused by the infection, and an increase in ALT levels was detected in N3-treated mice (Fig. 3A-B). N1 and N3 also presented renal toxicity by increasing the serum urea levels by approximately 25% (Fig. 3C). On the other hand, N1-treated animals showed similar serum CK-MB levels as the noninfected control animals, which represented a 45% reduction in comparison to those in the infected group. N2 and N3 did not alter the CK-MB levels (Fig. 3D).

The *in silico* predictive results of physicochemical descriptors, lipophilicity and water solubility of N1, N2 and N3 are shown in Table I. The parameters analysed include relative molecular mass, log P partition coefficient, number of hydrogen bond donors, number of hydrogen bond acceptors, topological polar surface area (TPSA), number of rotary bonds and aqueous solubility at a given pH (LogS). Lipinski’s rules were used to evaluate the drug-like properties. The MV of each of the three compounds was less than 500 g/mol. The number of hydrogen bond donors of the three compounds was less than five while the number of hydrogen bond acceptors was less than 10. The partition coefficient between n-octanol and water (log P_o/w_) is the classic lipophilicity descriptor. Different prediction methods (XLOGP3, WLOGP, MLOGP, SILICOS-IT, iLOGP) were used to reach the log P_o/w_ consensus.^20^ Consensus log P_o/w_ is the arithmetic mean of the values predicted by the five proposed methods. N1 was the only one with a consensus log P_o/w_ greater than five. N2 was the one with the highest TPSA, with 53.70 Å², while N1 and N3 obtained 37.91 Å² and 37.91 Å² respectively. However, a drug may be absorbed above 90% if the TPSA value is less than 60 Å². The number of rotary bonds of a candidate drug is also important for absorption capacity, and good absorption can be predicted when the number of rotary bonds is less than 10. All compounds had only one predicted rotatory bond. On the other hand, water solubility was measured using a topological method through the implementation of the ESOL model.^23^ The predicted values are the decimal logarithm of molar solubility in water (log S), with compound N3 being the only predicted compound with moderate solubility.

**TABLE I:** *In silico* predictive results of physicochemical descriptors, lipophilicity and water solubility of N1, N2 and N3 compounds

	N1	N2	N3
Physicochemical properties			
Molecular weight	342.43 g/mol	367.44 g/mol	328.41 g/mol
Nº rotatable bonds	1	1	1
Nº H-bond acceptors	2	2	2
Nº H-bond donors	1	2	1
TPSA	37.91 Å²	53.70 Å²	37.91 Å²
Lipophilicity			
Log Po/w (iLOGP)	3.48	3.00	3.26
Log Po/w (XLOGP3)	5.74	5.51	5.38
Log Po/w (WLOGP)	5.80	5.97	5.49
Log Po/w (MLOGP)	4.38	3.86	4.16
Log Po/w (SILICOS-IT)	6.33	6.33	5.82
Consensus Log Po/w	5.15	4.93	4.82
Water solubility			
Log S (ESOL)	-6.05	-6.10	-5.76
Class	Poorly soluble	Poorly soluble	Moderately soluble

**Fig. 4:**
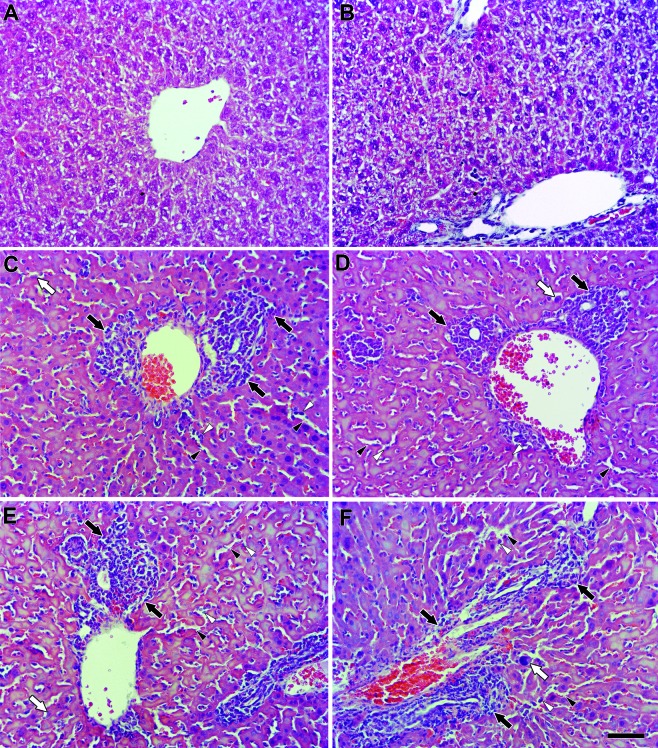
the effect of N1, N2 and N3 on the hepatic tissue of acute *Trypanosoma cruzi* infected mice. Representative hematoxylin and eosin (HE) stained sections from the (A, B) noninfected control, (C) infected, (D) N1, (E) N2 and (F) N3 groups. Black arrows: inflammatory foci. White arrows: Kupffer cells. Black arrowheads: sinusoidal dilatation. White arrowheads: inflammatory cells. Bar = 50 µM.

**Fig. 5:**
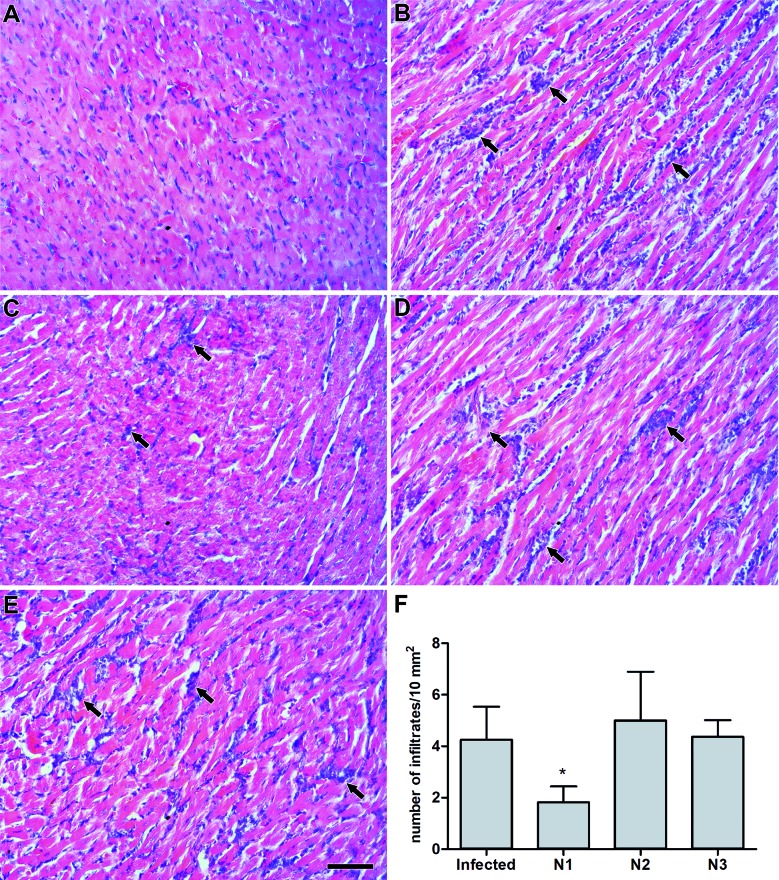
the effect of N1, N2 and N3 on cardiac tissue of acute *Trypanosoma cruzi* infected mice. (A-E) Representative hematoxylin and eosin (HE) stained sections from noninfected (A), infected (B), N1 (C), N2 (D) and N3 (E) mice. Arrows indicate inflammatory foci. Bar = 50 µM. (F) The number of inflammatory infiltrates. Data were expressed as the means + standard deviation (SD) from three independent experiments. *: comparisons between untreated and treated groups (p < 0.0001).

The histopathological analysis of the liver revealed a high number of inflammatory foci that were concentrated within periportal spaces in both untreated and treated infected animals (Fig. 4). Treatment with the three naphthoimidazoles led to sinusoidal dilatation with the presence of inflammatory cells inside the sinusoids and Kupffer cell proliferation (Fig. 4D-F). In the heart, the infection severely affected the tissue, and an intense inflammatory response was also frequently observed in noninfected animals (Fig. 5A-B). A largenumber of inflammatory infiltrates could still be detected after treatment with N2 and N3 (Fig. 5D-E). Only N1partially decreased cardiac inflammation, causing a 57% reduction in the number of infiltrates (Fig. 5C,F).

**Fig. 6:**
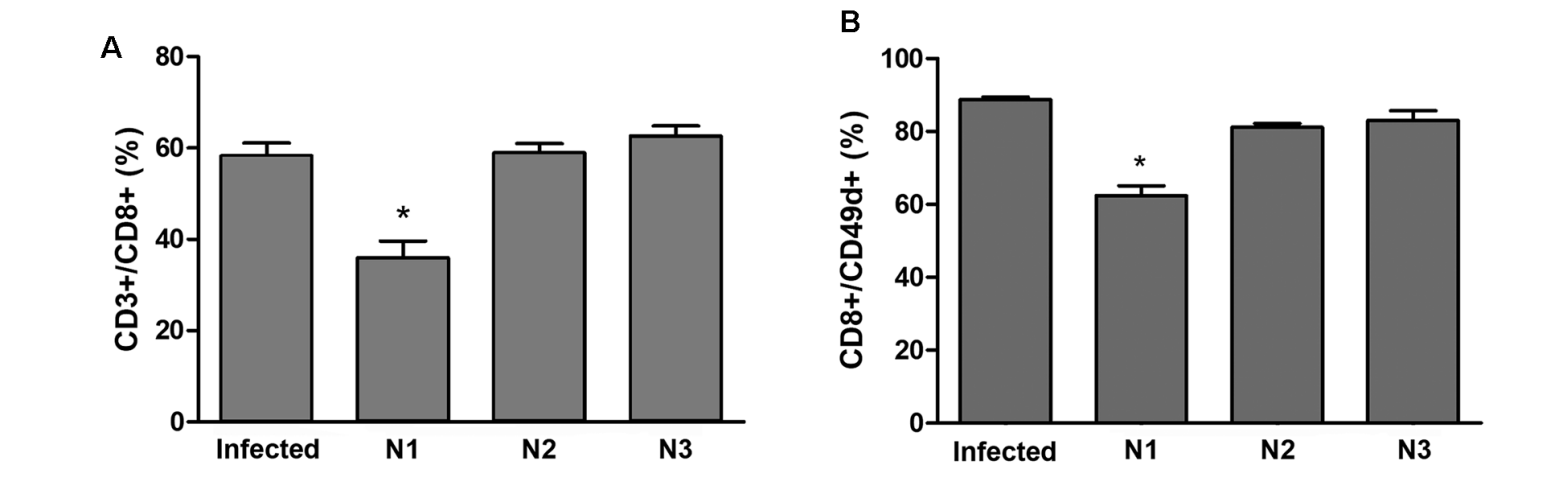
the effect of N1, N2 and N3 on cardiac inflammation in acute *Trypanosoma cruzi* infected mice. The hearts of nontreated infected mice (control) and N1-, N2- and N3-treated mice were collected at 15 dpi. Cardiac tissue was enzymatically dissociated, and all obtained cells were analysed by flow cytometry. (A, B) Mononuclear cells were gated according to the FSC x SSC parameters and analysed according to the expression of CD3/CD8 (A) and CD8/CD49d (B). Data were expressed as the means ± standard deviation (SD) from three independent experiments. *: comparisons between nontreated and treated groups (p < 0.05).

Since treatment with N1 promoted a reduction in the serum CK-MB levels and the number of inflammatory infiltrates in heart tissue, we decided to evaluate cardiac lymphocytic infiltration after treatment with the three naphthoimidazoles. This approach corroborated our biochemical and morphological results. A high percentage of cardiac CD8+ T lymphocytes (± 60%) was found in the infected control. Interestingly, a significant reduction of 38% in the population of cardiac CD8+ cells was observed only after N1 treatment (Fig. 6A). The splenocyte analysis revealed the reduced expression of the adhesion molecule CD49d on CD8+ T lymphocytes from N1-treated animals. No change in the expression of this molecule was observed in animals treated with N2 or N3 (Fig. 6B).The expression of the selectin CD62L and the integrins LFA-1 and CD49e were also evaluated in splenic CD8+ T lymphocytes, but no changes were observed in N1, N2 and N3 treated animals compared to untreated infected animals (data not shown).

**Fig. 7:**
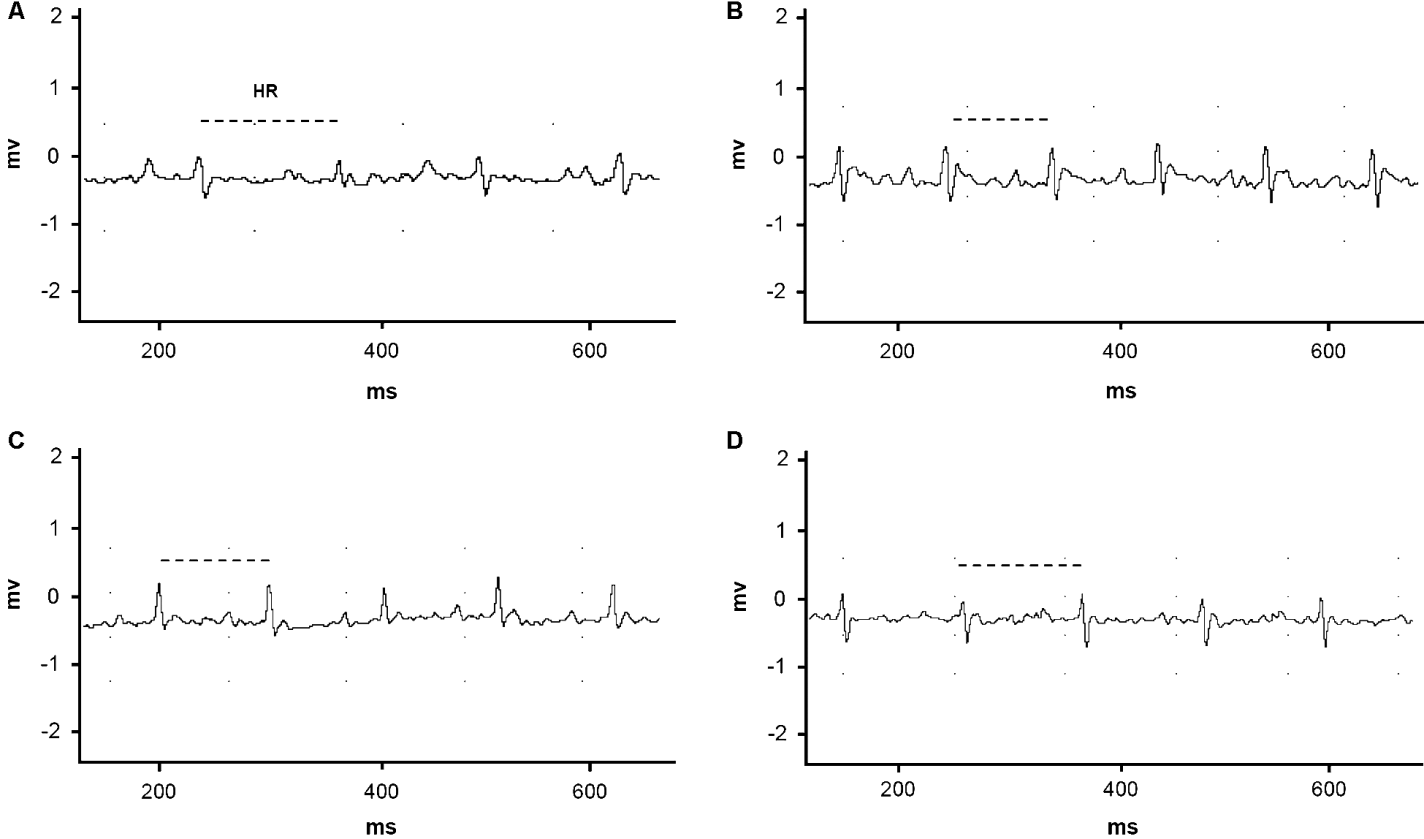
the effect of N1, N2 and N3 on cardiac electric activity in acute *Trypanosoma cruzi* infected mice. (A-D) ECG traces (14 dpi). (A) Infected control. (B) N1. (C) N2. (D) N3. HR: Heart rate. Dashed lines represent the time ranges for which the differences between the nontreated and treated groups were statistically significant (p ≤ 0.05).

ECG analysis revealed an increase of 176.5% in the PR interval, which was associated with a reduction of 37% in the heart rate of control animals at 14 dpi in comparison to that at 0 dpi. In relation to treatment, no significant differences between the naphthoimidazole-treated and nontreated groups were detected at up to 7 dpi (Table II, Fig. 7). At 14 dpi, sinus bradycardia was clearly observed in the infected and N3-treated groups, who had heart rates of 543 ± 52 and 562 ± 95 bpm, respectively. Treatment with N1 and N2 significantly improved this parameter, yielding heart rates of 640 ± 96 and 633 ± 69 bpm, respectively (Table II, Fig. 7). In parallel, the evaluation of blood pressure produced similar data for all experimental groups at all studied time points [Supplementary data (Fig. 3)].

**TABLE II:** Electrocardiographic (ECG) analysis of β-lapachone-derived naphthoimidazoles-treated mice

Group	n	PR interval (ms)	QRS interval (ms)	QTC interval (ms)	Heart rate (bpm)
dpi 0					
infected	6	24.3 ± 3.7	12.5 ± 2.3	31.5 ± 4.7	743 ± 56
N1	6	26.5 ± 7.0	12.8 ± 2.1	28.0 ± 3.3	799 ± 36
N2	6	27.3 ± 3.0	11.1 ± 1.4	28.4 ± 5.8	788 ± 76
N3	6	27.3 ± 6.5	10.9 ± 2.6	26.6 ± 5.8	769 ±146
dpi 7					
infected	6	29.6 ± 3.1	12.9 ± 3.3	27.2 ± 5.2	719 ± 71
N1	6	29.9 ± 2.7	11.4 ± 1.7	22.9 ± 4.3	741 ± 31
N2	6	28.7 ± 4.4	11.3 ± 1.1	27.1 ± 3.1	715 ± 72
N3	6	35.4 ± 16.0	10.8 ± 2.0	28.9 ± 5.7	725 ± 118
dpi 14					
infected	6	42.9 ± 5.4	10.4 ± 1.5	32.9 ± 14.3	543 ± 52
N1	6	39.0 ± 7.6	11.3 ± 2.1	26.9 ± 7.6	640 ± 96^*^
N2	6	42.1 ± 10.5	10.4 ± 2.8	28.9 ± 6.8	633 ± 69^*^
N3	6	45.3 ± 18.5	10.3 ± 1.7	27.0 ± 8.4	562 ± 95

Data were expressed as means + standard deviation (SD) from three independent experiments. *: comparisons between infected x treated groups (p ≤ 0.05).

## DISCUSSION

Despite their high efficacy in the acute phase of Chagas disease, the currently used drugs benznidazole and nifurtimox show important limitations, such as severe side effects and variability in activity, that depend on the parasite stock and/or disease stage.^24^ In 2015, a prospective, multicentric and randomised study demonstrated the limited effect of benznidazole on patients with established chronic infection, demonstrating that this drug did not reduce the progression of cardiomyopathy.^25^ There is an urgent need for efficient alternatives for the etiological treatment of Chagas disease. Diverse approaches have been employed, such as the continuous screening in *T. cruzi* of natural and synthetic substances from a great variety of chemical libraries. The development of new formulations of benznidazole and drug repurposing and/or combination are strategies that have been proposed for the treatment of parasitic illnesses, including Chagas disease.^5^

The bioactivity of naphthoquinones such as β-lapachone, lapachol and C-allyl lawsone have been extensively described, and *in vitro* trypanocidal activity has also beendemonstrated.^8^ Since 1997, our group has been investigating the anti-*T. cruzi* effect of β-lapachone derivatives on infective bloodstream trypomastigotes, and we have found that the naphthoimidazoles N1, N2 and N3 are the most active.^9^ ^,^ ^10^ ^,^ ^11^ Nevertheless, information about the biological effects of naphthoimidazoles is scarce. Recently, the antibacterial and anti-inflammatory activities of naphthoimidazoles were published.^26^ For protozoa, all previous reports were authored by our group. Mechanistic studies revealed the activity of these naphthoquinone derivatives against all *T. cruzi* stages *in vitro* and revealed the mitochondrion as the main target.^12^ ^,^ ^13^ Such mitochondrial susceptibility was confirmed by proteomic approaches, which also showed that trypanothione synthetase overexpression was induced by treatment.^15^ ^,^ ^16^ Recently, we further investigated the mechanism of action of naphthoimidazoles, which revealed the oxidative misbalance derived from the direct effect on the mitochondrial electron transport chain (at least for N1) that leads to the production of high ROS levels to kill the parasite.^17^ Other mechanistic proposals could not be discarded, especially in terms of explaining the action of N2 and N3.

The present work is the first *in vivo* evaluation of naphthoimidazoles described in the literature. According to the previously proposed guidelines,^27^ we analysed their effect on a murine acute model of Chagas disease. The compounds were administered by gavage in five doses at a dosage of 100 mg/kg body weight every other day starting at 7 dpi after parasite detection in the bloodstream. In the control group, the parasitaemia curve displayed atypical profile, with the highest peak occurring at 8 dpi and an increase in the mortality rate occurring at 15 dpi. These data, together with results showing high serum levels of hepatic and cardiac enzymes and other electrocardiographic and immunophenotypic findings, validate our acute model.^21^ Naphthoimidazoles interfered with the infection course, reducing the parasitaemia peak; however, unfortunately, no protection was observed in terms of the mortality rates. A noninvasive analysis also showed that nontreated and treated animals shared similar patterns in terms of feeding, motor and exploratory activities, indicating that there was no clinical improvement due to the treatment.

Based on the parasitaemia measurements, N3 was the most efficient drug, as inhibited the number of circulating parasites, but it was also the most toxic derivative. Hepatic (ALT) and renal levels were significantly higher in the N3-treated group than in the infected controls, indicating the presence of injuries in both organs that were associated with the intense spinning motion suggestive of vestibular or neurological damage; this led to the exclusion of N3 from subsequent evaluation. Treatment with N2 improved only two parameters in our model by partially reducing parasitaemia and bradycardia, while the biochemical and behavioral analyses indicated a pattern similar to that of the untreated infected group. Together, these results indicate that the absence of an effect on the mortality of the animals shows that N2 is not a promising candidate for chronic model studies.

More than twenty years after the first report on N1 trypanocidal activity,^12^ the N1 derivative stands out as the most promising β-lapachone-derived naphthoimidazole. Although our results showed only a discrete reduction in parasitaemia (approximately 25%), no difference in the mortality rates and increased urea levels, N1 led to the dramatic recovery of acute cardiomyopathy. This singularity of N1 could be, at least partially, explained by our *in silico* data, that points to high lipophilicity (log P_o/w_ > 5), showing a distinct chemical behavior from two other compounds. N1 reversed the increase in CK-MB caused by the infection, significantly reduced the number of myocardial inflammatory infiltrates, decreased the population of cardiac CD8+ T cells associated with myocarditis progression in both *T. cruzi* acute and chronic infections, and reduced the percentage of CD8+/CD49d+ T lymphocytes in the spleen.^28^ CD49d is an integrin that acts as an important adhesion molecule during the migration of blood lymphocytes to target tissues. The reduction in the percentage of CD8+/CD49d+ T lymphocytes may, in part, be responsible for the decreased migration of these lymphocytes to the heart in N1-treated animals. N1 also partially reverted bradycardia, although no improvement in other ECG parameters was observed. The present results, together with data in previous literature, encourage us to conduct further immunological studies aimed at decreasing cardiac inflammation in animal models. The use of anti-arrhythmic drugs such as amiodarone combined with beta-blockers (atenolol and propanolol) and/or pacemakers has been extensively described for the treatment of heart dysfunction.^29^ By focusing on myocarditis reduction, immunomodulatory approaches aimed at reversing heart disease in experimental Chagas model shave been proposed, which may lead to new methods for the management of cardiac patients; immunomodulatory drugs could represent an interesting strategy for this.^30^ Alternative N1 formulations and their combination with trypanocidal and/or anti-arrhythmic drugs could be tested, which may reduce the naphthoimidazole dose and consequently its toxicity. Such combinations could be attractive option for the treatment of chronically infected individuals; however, further pharmacological tests and other experimental analyses must be performed before clinical trials are conducted.

## References

[1] World Health Organization (2015). Chagas disease in Latin America: an epidemiological update based
on 2010 estimates.

[2] Alonso-Padilla J, Cortés-Serra N, Pinazo MJ, Bottazzi ME, Abril M, Barreira F (2019). Strategies to enhance access to diagnosis and treatment for
Chagas disease patients in Latin America. Expert Rev Anti Infect Ther.

[3] Messenger LA, Bern C (2018). Congenital Chagas disease current diagnostics, limitations and
future perspectives. Curr Opin Infect Dis.

[4] Coura JR, Borges-Pereira J (2011). Chronic phase of Chagas disease why should it be treated? A
comprehensive review. Mem Inst Oswaldo Cruz.

[5] Salomao K, Menna-Barreto RFS, De Castro SL (2016). Stairway to heaven or hell Perspectives and limitations of chagas
disease chemotherapy. Curr Top Med Chem.

[6] Hussain H, Green IR (2017). Lapachol and lapachone analogs a journey of two decades of patent
research (1997-2016). Exp Opin Ther Pat.

[7] Anaissi-Afonso L, Oramas-Royo S, Ayra-Plasencia J, Martín-Rodríguez P, García-Luis J, Lorenzo-Castrillejo I (2018). Lawsone, juglone, and ß-lapachone derivatives with enhanced
mitochondrial-based toxicity. ACS Chem Biol.

[8] Pinto AV, De Castro SL (2009). The trypanocidal activity of naphthoquinones A
review. Molecules.

[9] Pinto AV, Pinto CN, Pinto MDCFR, Rita RS, Pezzella CAC, De Castro SL (1997). Trypanocidal activity of synthetic heterocyclic derivatives of
active quinones from Tabebuia sp. Arzneimittel-Forschung/Drug Res.

[10] Pinto CN, Dantas AP, De Moura KCG, Emery FS, Polequevitch PF, Pinto MCFR (2000). Chemical reactivity studies with naphthoquinones from Tabebuia
with anti-trypanosomal efficacy. Arzneimittel-Forschung/Drug Res.

[11] De Moura KCG, Salomão K, Menna-Barreto RFS, Emery FS, Pinto MDCFR, Pinto AV (2004). Studies on the trypanocidal activity of semi-synthetic
pyran[b-4,3] naphtho[1,2-d]imidazoles from ß-lapachone. Eur J Med Chem.

[12] Menna-Barreto RFS, Henriques-Pons A, Pinto AV, Morgado-Diaz JA, Soares MJ, De Castro SL (2005). Effect of a ß-lapachone-derived naphthoimidazole on Trypanosoma
cruzi identification of target organelles. J Antimicrob Chemother.

[13] Menna-Barreto RFS, Corrêa JR, Pinto AV, Soares MJ, De Castro SL (2007). Mitochondrial disruption and DNA fragmentation in Trypanosoma
cruzi induced by naphthoimidazoles synthesized from
ß-lapachone. Parasitol Res.

[14] Menna-Barreto RFS, Beghini DG, Ferreira ATS, Pinto AV, De Castro SL, Perales J (2010). A proteomic analysis of the mechanism of action of
naphthoimidazoles in Trypanosoma cruzi epimastigotes in
vitro. J Proteomics.

[15] Brunoro GVF, Faça VM, Caminha MA, Ferreira ATS, Trugilho M, de Moura KCG (2016). Differential gel electrophoresis (DIGE) evaluation of
naphthoimidazoles mode of action a study in Trypanosoma cruzi bloodstream
trypomastigotes. PLoS Negl Trop Dis.

[16] Bombaça ACS, Viana PG, Santos ACC, Silva TL, Rodrigues ABM, Guimarães ACR (2019). Mitochondrial disfunction and ROS production are essential for
anti-Trypanosoma cruzi activity of ß-lapachone-derived
naphthoimidazoles. Free Radic Biol Med.

[17] Brener Z (1962). Therapeutic activity and criterion of cure on mice experimentally
infected with Trypanosoma cruzi. Rev Inst Med Trop São Paulo.

[18] da Silva DR, de Castro SL, Alves MCS, Batista WS, de Oliveira GM (2012). Acute experimental Trypanosoma cruzi infection establishing a
murine model that utilises non-invasive measurements of disease
parameters. Mem Inst Oswaldo Cruz.

[19] Campos JDS, Hoppe LY, Duque TLA, de Castro SL, Oliveira GM (2016). Use of noninvasive parameters to evaluate Swiss webster mice
during Trypanosoma cruzi experimental acute infection. J Parasitol.

[20] Daina A, Michielin O, Zoete V (2017). SwissADME a free web tool to evaluate pharmacokinetics,
drug-likeness and medicinal chemistry friendliness of small
molecules. Sci Rep.

[21] de Oliveira GM, Masuda MO, Rocha NN, Schor N, Hooper CS, de Araújo-Jorge TC (2009). Absence of Fas-L aggravates renal injury in acute Trypanosoma
cruzi infection. Mem Inst Oswaldo Cruz.

[22] Mitchell GF, Jeron A, Koren G (1998). Measurement of heart rate and Q-T interval in the conscious
mouse. Am J Physiol.

[23] Delaney JS (2004). ESOL estimating aqueous solubility directly from molecular
structure. J Chem Inf Comput Sci.

[24] Filardi LS, Brener Z (1987). Susceptibility and natural resistance of Trypanosoma cruzi
strains to drugs used clinically in Chagas disease. Trans R Soc Trop Med Hyg.

[25] Morillo CA, Marin-Neto JA, Avezum A, Sosa-Estani S, Rassi A, Rosas F (2015). Randomized trial of benznidazole for chronic chagas'
cardiomyopathy. N Engl J Med.

[26] Abraham R, Prakash P, Mahendran K, Ramanathan M (2018). A novel series of N-acyl substituted indole-linked benzimidazoles
and naphthoimidazoles as potential anti inflammatory, anti biofilm and anti
microbial agents. Microb Pathog.

[27] Romanha AJ, de Castro SL, Soeiro MNC, Lannes-Vieira J, Ribeiro I, Talvani A (2010). In vitro and in vivo experimental models for drug screening and
development for Chagas disease. Mem Inst Oswaldo Cruz.

[28] Silverio JC, Pereira IR, Cipitelli MC, Vinagre NF, Rodrigues MM, Gazzinelli RT (2012). CD8+ T-cells expressing interferon gamma or perforin play
antagonistic roles in heart injury in experimental Trypanosoma
cruzi-elicited cardiomyopathy. PLoS Pathog.

[29] Pereira-Barretto AC, Bacal F, de Albuquerque DC (2015). Most heart failure patients die from pump failure implications
for therapy. Am J Cardiovasc Drugs.

[30] Vilar-Pereira G, Pereira IR, Ruivo LAS, Moreira OC, da Silva AA, Britto C (2016). Combination chemotherapy with suboptimal doses of benznidazole
and pentoxifylline sustains partial reversion of experimental Chagas' heart
disease. Antimicrob Agents Chemother.

[31] Lopes JN, Cruz FS, Docampo R, Vasconcellos ME, Sampaio MC, Pinto AV (1978). In vitro and in vivo evaluation of the toxicity of
1,4-naphthoquinone and 1,2-naphthoquinone derivatives against Trypanosoma
cruzi. Ann Trop Med Parasitol.

