# Supplementary material for: Antiparasitic and anti-inflammatory activities of ß-lapachone-derived naphthoimidazoles in experimental acute Trypanosoma cruzi infection
Source: Mem Inst Oswaldo Cruz. 2020 Feb 14;115:e190389. doi: 10.1590/0074-02760190389 (PMC7029714; doi:10.1590/0074-02760190389)
Supplement: Supplementary file 1 [file 1678-8060-mioc-115-e190389-s.pdf]

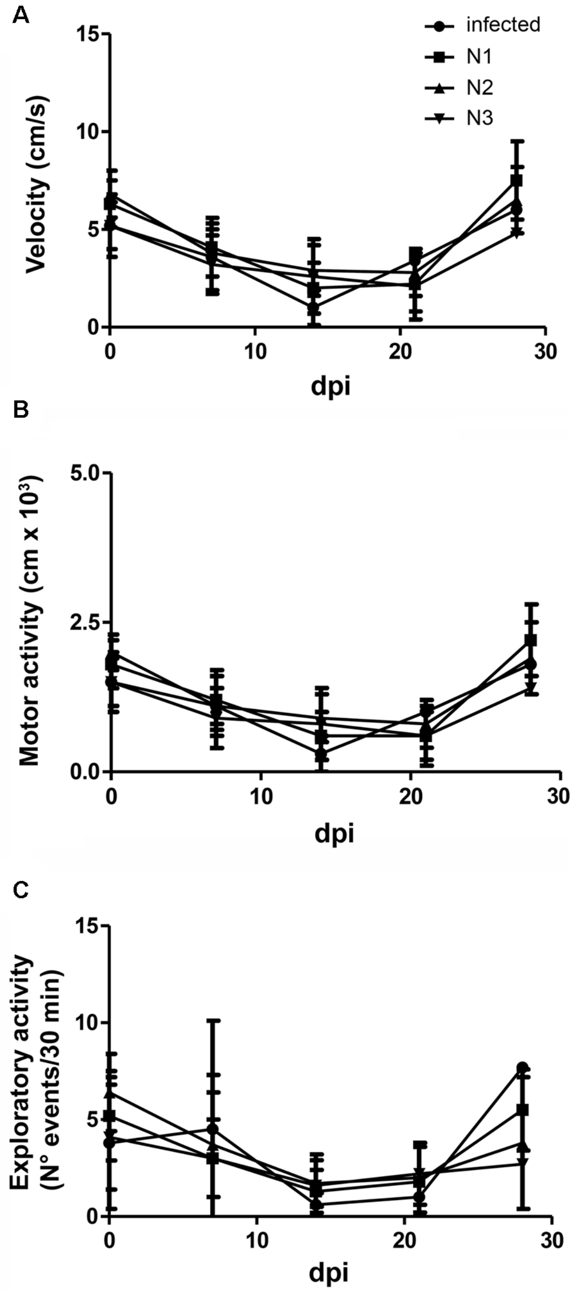

Fig. 1: the effect of N1, N2 and N3 on the behavior of acute *Trypanosoma cruzi* infected mice. (A) Velocity, (B) motor and (C) exploratory activities.

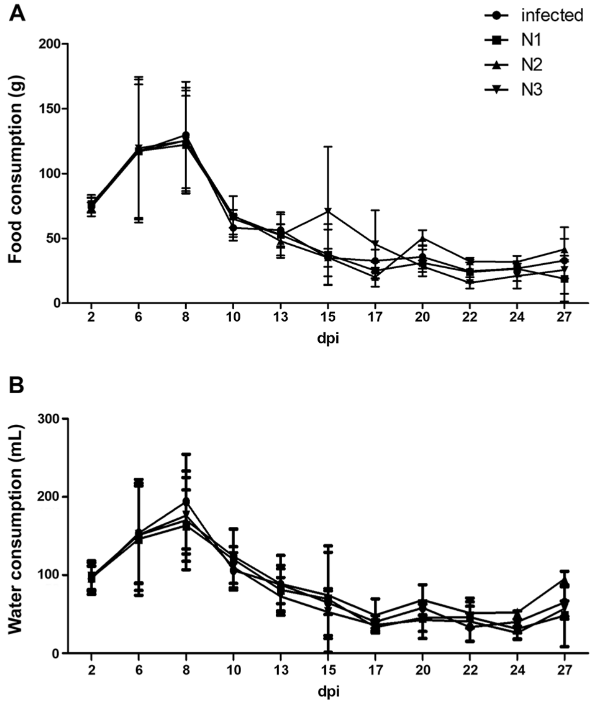

Fig. 2: the effect of N1, N2 and N3 on the food and water consumption of acute *Trypanosoma cruzi* infected mice. (A) Food consumption. (B) Water consumption.

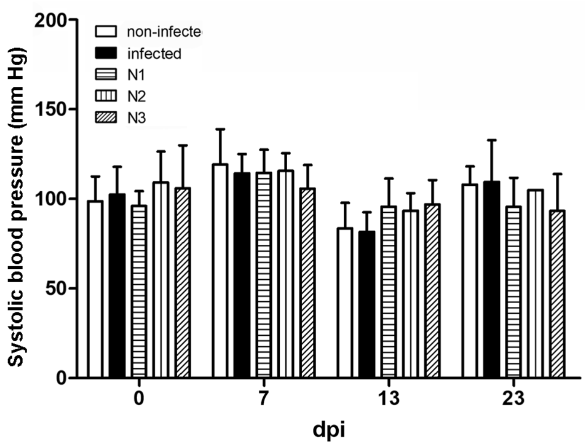

Fig. 3: the effect of the naphthoimidazoles N1, N2 and N3 on the blood pressure of acute *Trypanosoma cruzi* infected mice. (A) Food consumption. (B) Water consumption.
